# The Experience-Experience Gap: Distributional Learning Is Associated with a Divergence of Preferences from Estimations

**DOI:** 10.21203/rs.3.rs-6282612/v1

**Published:** 2025-04-10

**Authors:** Boaz Rosenberg, Eran Eldar

**Affiliations:** 1 Department of Psychology, Hebrew University of Jerusalem, Jerusalem, Israel; 2 Cognitive and Brain Sciences Departments, Hebrew University of Jerusalem, Jerusalem, Israel

## Abstract

Recent landmark studies show that the brain is equipped to learn not just average expected outcomes, but entire distributions of expected outcomes. Yet the role of such distributional learning in shaping human decision-making remains to be determined. To study this question, we designed two tasks where participants experienced different outcome distributions, provided their estimates of each, and reported their preferences among them. In one task, which facilitated distributional learning, participants’ preferences significantly diverged from their own estimates, consistent with predictions of Prospect Theory. Conversely, in a task that hindered distributional learning, the divergence of preferences from estimates was eliminated. Computational modelling showed how distributional learning may be responsible for disassociating preferences from estimations by enabling the application of a utility function to different potential outcomes. Our findings offer a new understanding of when and how preferences deviate from normative decision-making, a fundamental question in the study of human rationality.

Distributional reinforcement learning, which equips agents with representations of entire outcome distributions rather than single point estimates, has recently gained prominence in our understanding of learning from experience. Following the development of distributional learning algorithms by computer scientists^1–3^, studies of neural function have found evidence for their possible implementation in rodents^4,5^ and primates^6^. Additional studies suggest that humans, too, may learn distributions of potential outcomes. Such studies found that human brain function is sensitive to stimulus and outcome distributions^7,8^ and is well suited to implement distributional learning^9^. However, the critical question of how learning distributions of outcomes associated with different choices shapes preferences among them has yet to be investigated.

This question is pertinent because well-known theories of decision-making suggest that distributional learning may exert a profound impact on human preferences. This is particularly the case with regards to prospect theory^10^, which posits that people form preferences by using a non-linear utility function that translates objective outcomes into subjective values. For example, winning $100 does not have twice the value of winning $50; rather, it is often valued less due to a utility function that entails diminishing marginal returns in the domain of gains. This creates preferences for or against risk, defined as outcome variance, even when the expected outcomes of potential choices are equal (i.e., a certain $50 is preferred over a 50% of receiving $100). This is because preferences are not guided by expected outcomes, but rather by expected utilities. Expected utility, however, cannot be calculated from summary statistics alone. Due to its non-linearity, the utility function needs to be separately applied to each possible outcome, and only then can the different utilities be averaged into an expected utility.

An agent that maximizes expected utility, therefore, must either know the full distribution of outcomes, or convert each observed outcome into a utility already at the time of observation and thus directly learn the expected utility (e.g., via Rescorla-Wagner updates^11^). Whereas the second approach is computationally simpler and does not require distributional learning, it makes it impossible for agents to adapt their utility calculations after learning. Such adaptation may be required because the utility function’s reference point, which determines whether an outcome is perceived as a gain or a loss, changes over time^12^. Distributional learning thus offers a plausible and adaptable way to implement prospect-theory-congruent decision making.

Indeed, the prescriptions of prospect theory have primarily been confirmed in experiments where people were given full descriptions of available outcome distributions. In contrast, when learning from experience, people often exhibit significantly diminished or even reversed risk preferences^13–15^. This inconsistency between the two types of decision-making has come to be known as the “description–experience gap”. Several factors have been suggested to contribute to this gap, among which are memory biases^16^ and a reliance on small samples of recent outcomes when learning from experience^17^. Importantly, memory of the frequencies of particular outcomes could reflect a degree of distributional learning, whereas a reliance on recent outcomes may reflect the operation of common non-distributional learning models, which use constant learning rates and thus overweight recent experiences. Distributional learning, or its absence, may thus play a crucial role in shaping experience-based preference formation.

To investigate the effects of distributional learning on human preferences, we designed an experiment where participants experienced various outcomes under conditions that either facilitated or hindered their ability to learn how outcomes were distributed. We assessed participants’ detailed estimates of the outcome distributions and their objective expected values. Additionally, we recorded participants’ choice-based and emotional preferences among the distributions. Strikingly, we found that preferences deviated from participants own estimations, as prescribed by prospect theory, only in conditions that facilitated learning of outcome distributions. We then used computational modeling to show that these effects are best explained by a model wherein distributional learning enables the application of a utility function to the different potential outcomes. These findings reveal a key role for distributional learning in shaping human preferences and offer a novel mechanism explaining deviations from normative models of decision making.

## Results

### Humans successfully learn distributions from experience

**Figure 1.**
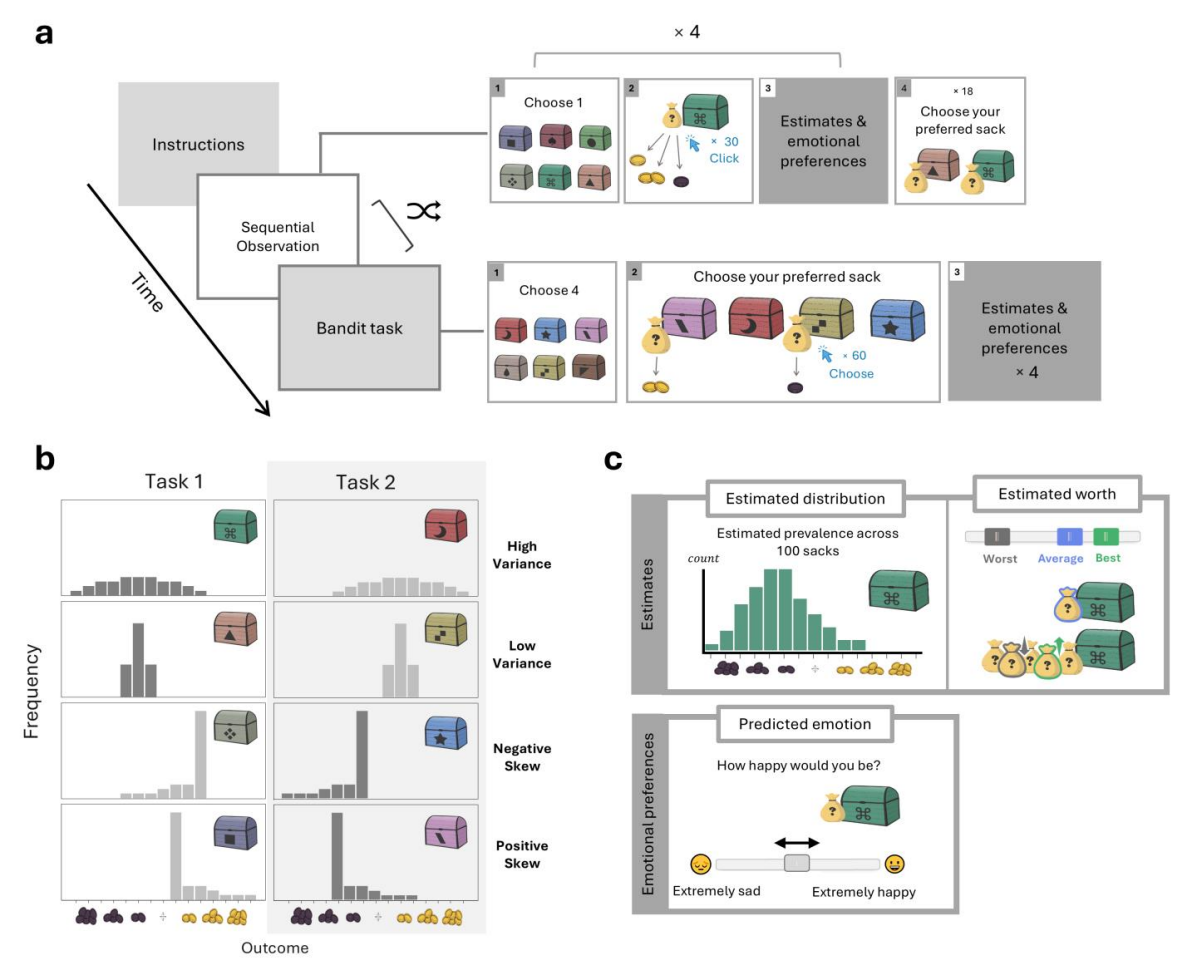
Experimental design. **a. Task layout.** The study comprised two tasks: a Sequential Observation (SO) task and a bandit task. In the SO task, participants observed the contents of 30 sacks, drawn one at a time from a chest, separately for each chest. In the bandit task, participants observed the same exact outcomes drawn from four new chests but in an interleaved manner as part of a bandit task. In both tasks, participants reported their estimates and preferences concerning each of the chests. Task order was randomized across participants. **b. Outcome distributions.** Each of the four chests in both tasks was assigned one of four distributions: normal with high variance, normal with low variance, positively skewed, or negatively skewed. The mean of each kind of distribution was manipulated by creating two versions of the task, one where the normal distributions had a positive mean and the skewed distributions a negative mean, and one with the opposite mapping. These versions were randomly assigned to either the SO or bandit task for each participant. Positive outcomes were represented by gold coins and negative outcomes by dark coins. **c. Estimations and emotional preferences.** In both tasks, in addition to choosing between chests, participants provided for each chest two kinds of estimates and one measure of emotional preference. The two estimates comprised the number of times participants expected to receive each outcome if 100 additional sacks were drawn from the chest (‘estimated distribution’), and the expected number of gold or dark coins of a typical single sack, as well as of the best and worst sacks out of five drawn from the chest (‘estimated worth’). The emotional preference measure consisted of participants’ predicted emotional responses to being rewarded based on the outcomes of a sack drawn from that chest.

To facilitate distributional learning, we designed a sequential observation (SO) task where participants sequentially observed outcomes drawn from a given distribution. In each round, participants observed the number of gold coins found in different sacks drawn from a particular treasure chest. Each sack contained up to 7 coins that were either all gold, denoting gain, or all dark, denoting loss. Possible outcomes thus ranged from −7 to +7. The sacks were drawn from the chest one after the other, and participants were instructed to pay attention to their distribution. After observing the contents of 30 sacks, participants were asked to report their objective estimates and subjective preferences regarding the sacks in the chest. This procedure was repeated four times, each time using a new chest with a distinct distribution of sacks. These distributions were designed to vary in mean, variance, and skewness to enable testing participants’ sensitivity to each of the first three moments of the distributions (Figure 1b).

**Figure 2.**
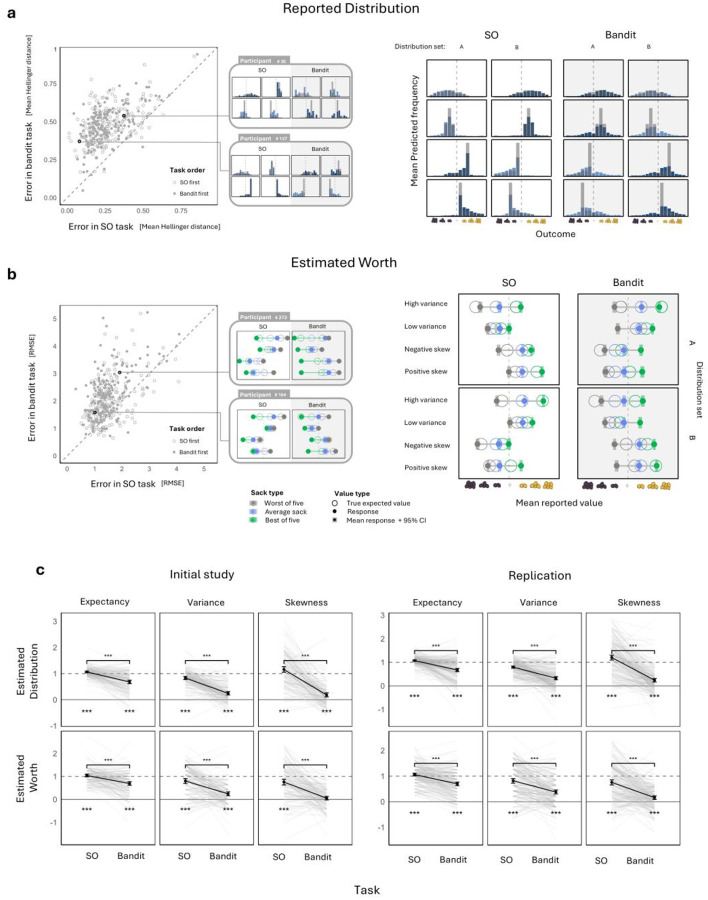
Accuracy and sensitivity of participants’ estimates. **a. Estimated distributions.** The left panel shows estimation errors per participant in both tasks, measured as the mean Hellinger distance between reported and actual distributions. Participants lying above the diagonal identity line were more accurate in the SO task than in the bandit task. Estimates (colored histograms) versus true distributions (gray histograms) are shown for two example participants, and for group average estimates (right panel). Axes labels are omitted in the smaller exemplary plots, but are the same across all plots. **b. Estimated worths.** The left panel shows estimation errors measured as the Root Mean Square Error (RMSE). Estimates (filled circles) versus actual (empty circles) worths are shown for two example participants, and for group average estimates (right panel). **c. Sensitivity to distribution moments.** Sensitivity of estimated distributions and worths to the mean, variance, and skewness of outcome distributions in both tasks. Sensitivity was calculated as the difference in the estimated distribution statistic for chests high and low on it, normalized such that a score of 1 indicates a difference in estimates that equals the true difference. All sensitivities where higher in the SO task as compared to the bandit task (***: p < 0.001; chance level = 0). Each line represents an individual participant. Black points represent the mean sensitivity across all participants. Error bars: 95% CI.

Participants reported their estimates in two ways. The first, termed “estimated distribution,” required participants to predict the number of times each possible outcome would occur if 100 sacks were drawn from the chest (Figure 1c). Overall, participants reported the distributions fairly accurately (Hellinger distance: 0.27 ± 0.007 (M ± SEM), chance level = 0.765; Figure 2a). To determine the sensitivity of participants’ estimates to the first three moments of the distributions, we treated the estimates as probability mass functions describing the outcome distributions. This enabled us to infer participants’ estimated mean, variance, and skewness for each chest. We then calculated the difference in each inferred statistic between chests where the statistic was high versus low, thus generating a sensitivity measure of participants’ learning to each statistical property of the outcome distributions.

We found that participants’ estimates were sensitive to all three statistics (all p values < 0.001, single sample t test), with average sensitivities deviating by no more than 20% from those of an ideal observer (Figure 2c). We replicated these results, along with all key subsequent results, in an additional preregistered study (all p values < 0.001; for a detailed report see Supplementary Table 1).

To determine whether participants were able to translate their knowledge of the distributions into calculations of expected outcome, we employed a second objective estimation measure, termed “estimated worth.” Here, we asked participants to estimate the average number of coins in an average sack drawn from the chest, as well as in the best and worst sacks out of five sacks drawn (Figure 1c). To ensure that participants’ answers reflected their objective estimates, and limit the influence of participants’ subjective evaluations, we specifically instructed participants to estimate the objective expected value of each sack and informed them that they would be rewarded based on the accuracy of their estimates relative to these objective values. Furthermore, we had participants complete a training round wherein we showed participants the sacks’ true expected values as exemplary correct responses.

Participants’ estimates of worth were also fairly accurate (RMSE: 1.55 ± 0.03, chance level = 4.16; Figure 2b). To compute these estimates’ sensitivity to the three first moments of each distribution, we treated the estimated worth of a single sack as an estimate of distribution mean, the difference between the worths of the best and worst sacks out of five as a correlate of distribution variance, and the ratio between the deviations of the best and worst sacks from the single sack as a correlate of estimated skewness. We then examined the differences in these measures between chests with distributions that were high or low on each statistic. Here too we found that participants’ estimated worths were sensitive to all three statistics (single sample t test, all p values < 0.001 in both initial study and replications; supplementary table 1), with the average sensitivity deviating by no more than 25% from that of an ideal observer.

**Figure 3:**
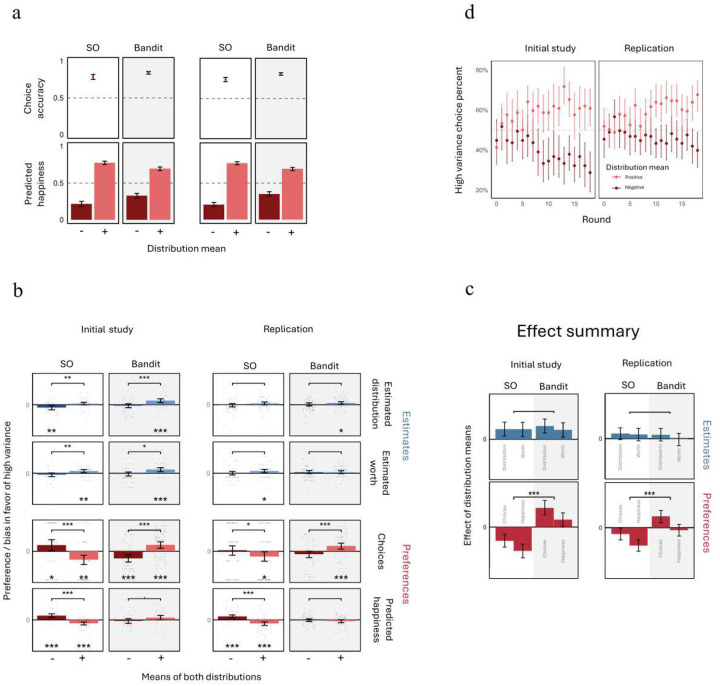
Risk preferences and corresponding estimation biases. **a. Preferences as a function of mean outcome:** Preferences are shown as mean choice rate and mean happiness rating. Error bars represent 95% confidence intervals (CIs). The results confirm that participants preferred chests with higher expected outcomes. **b. Risk preferences and corresponding estimation biases:** This panel shows mean estimation biases and preferences favoring high, as opposed to low, variance across different measures. The data were split by task and by whether the two distributions’ mean was positive or negative. Data were z-scored for comparability across measures. Error bars represent 95% CIs. Points represent individual participants. Asterisks below columns denote significant difference from 0, whereas brackets with asterisks indicate significance of difference between columns (*: p < 0.05; **: p < 0.01; ***: p < 0.001). **c. Effect summary:** This panel summarizes the results shown in panel b, quantifying the effect of outcome mean on risk preferences and corresponding estimation biases. This reveals a clear divergence in risk preferences between tasks, while estimation biases remain similar. Error bars represent 95% CIs. **d. Risky choice preferences in the bandit task over time:** This panel depicts the rate of choices favoring high-variance chest by round number in the bandit task. Error bars represent 95% CIs. As the task progressed, participants increasingly favored the high-variance option among positive-mean distributions, and the low-variance option among negative-mean distributions. Thus, prospect-theory-incongruent risk preferences strengthened over time.

In order to identify biases in participants’ estimations that might contribute to risk preferences, we compared their estimated means for chests with high versus low outcome variance. Since risk aversion is typically expected in the domain of gains, and risk seeking in the domain of losses, we tested whether differences in estimation biases for chests with high versus low variance depended on whether outcomes were primarily gains (chests with a positive mean outcome) or losses (chests with a negative mean outcome). We found such a moderation in the initial study (estimated distribution: $t_{\left(156\right)}=2.55$, p = 0.005; estimated worth: $t_{\left(127\right)}=2.74$, p = 0.007; Figure 3), but this moderation did not fully replicate (estimated distribution: $t_{\left(209\right)}=1.63$, p = 0.105; estimated worth: $t_{\left(167\right)}=1.29$, p = 0.2; Figure 3; see Supplementary Table 2 for full results). Most importantly, the observed moderation ran counter to prospect theory. That is, estimates favored high-variance chests to a greater extent among chests with a positive, as compared to a negative, mean.

Thus, participants successfully learned the outcome distributions in the SO task, and learning biases did not have the capacity to explain risk preferences in accordance with prospect theory.

### Interleaving observations impairs distributional learning

To determine the effects of distributional learning on human preferences, we had participants complete an additional task that was designed to hinder distributional learning (with task order randomized across participants). This task was a modified four-armed bandit task, where participants observed the outcomes of sacks drawn from four new treasure chests (Figure 1a, ‘bandit task’). This task provided participants with precisely the same observations as the SO task, but instead of showing all outcomes drawn from a given chest consecutively, outcomes drawn from different chests were interleaved. We believed this would decrease participants’ ability to learn the entire distributions of outcomes for each chest, thus making them resort to learning lower-dimensional summary statistics. After the bandit task, participants reported their estimations for all four chests in the same way as in the SO task.

As expected, estimated distributions (Hellinger distance: 0.48 ± 0.006) and estimated worths (RMSE = 2.15 ± 0.04) were substantially less accurate in the bandit, as compared to the SO, task (Figure 2a,b). Similarly, the sensitivity of both kinds of estimates to the first three moments of the distributions was strongly reduced (paired sample t test, all p-values < 0.001 in initial study and replication; Supplementary Table 3). This was especially the case with regards to distribution variance and skewness, for which learning sensitivity was at most 30% of that of an ideal observer (Figure 2c). To rule out alternative interpretations, we repeated the sensitivity reduction analysis using linear regression controlling for either task order or chest reporting order in the bandit task (where chests were estimated in bulk) and obtained the same results (all p-values <0.001, Supplementary Tables 4 and 5). These results confirm that distributional learning was severely limited in the bandit task.

Estimation biases in the bandit task were similar to those observed in the SO task (Figure 3). That is, in the initial study, biases concerning high- versus low-variance chests were moderated by whether outcomes where primarily gains or losses, counter to the prescriptions of prospect theory (independent samples t tests; estimated distribution: $t_{\left(152\right)}=3.67,\;p<0.001$; estimated worth: $t_{\left(147\right)}=2.64,\;p=0.009$), but this moderation did not replicate (estimated distribution: $t_{\left(209\right)}=1.27,\;p=0.2$; estimated worth: $t_{\left(205\right)}=0.1,\;p=0.92$). Thus, the bandit task successfully reduced distributional learning without introducing estimation biases that could influence risk preferences.

### A task-dependent deviation of preferences from estimations

To determine how the facilitation or hindrance of distributional learning affected participants’ risk preferences, we first examined their choices across both tasks. As expected, participants consistently selected chests with higher outcome means. Accuracy was slightly lower in the SO task (initial study: 79%, replication: 76%) compared to the bandit task (initial: 84% replication: 83%; Figure 3a), likely because choices in the SO task were made some time after the first chests’ outcomes were observed. However, in both tasks, accuracy was satisfactory and well above chance (p < 0.001; Supplementary Table 6). Risky choices, in contrast, were qualitatively different in the two tasks.

In the SO task, participants chose in line with prospect theory. That is, participants chose low-variance over high-variance chests more often when outcomes were primarily in the domain of gains, as compared to the domain of losses (independent samples t tests; $t_{\left(147\right)}=5.5,\;p<0.001$; replication: $t_{\left(208\right)}=1.99,\;p=0.048$; Figure 3b). The same pattern was present also in our second, emotion-based measure of preferences. After participants observed all outcomes drawn from each particular chest, we had them predict how happy they would be if their compensation was determined by the content of a sack randomly drawn from the chest, with each coin representing a gain or loss of $100 (Figure 1c). As expected, happiness ratings were substantially higher for chests with a positive, as compared to a negative, mean outcome (paired t tests; all p values < 0.001, in both initial and replication studies; Figure 3a, Supplementary Table 6). And in comparing low vs high variance chests, we again found a prospect-theory-congruent pattern of preferences (independent samples t tests; $t_{\left(131\right)}=7,\;p<0.001$; replication: $t_{\left(202\right)}=6.3,\;p<0.001$), consistent with participants’ choices.

These risk preferences in the SO task qualitatively deviated from participants’ own estimates and therefore could not be explained by biases in the learning process. To test this quantitatively, we normalized all preference and estimation bias scores comparing between low and high variance chests by scaling them based on their standard deviation. We then tested the difference between preferences and estimates in terms of the effect of chests’ mean outcome (positive vs negative). For all pairs of estimate and preference measures, the results confirmed a significant difference between estimation biases and preferences in the SO task (independent samples t tests; all p-values < 0.001 in both initial and replication studies, Supplementary Table 7).

By contrast, in the bandit task, participants’ choices showed the **opposite** risk preferences, choosing more high-variance chests among chests with a positive mean, and more low-variance chests among chests with a negative mean (independent samples t tests, initial: $t_{\left(147\right)}=-5.44,\;p<0.001$; replication: $t_{\left(197\right)}=-3.68,\;p<0.001$; Figure 3b). This reversal was reflected in a strong and reliable difference between tasks in the effect of mean outcome on risky choice preferences (independent samples t tests, initial: $t_{\left(148\right)}=6.5,\;p<0.001$; replication: $t_{\left(196\right)}=3.79,\;p<0.001$). Moreover, happiness ratings showed the same influence of task on risk preferences (independent samples t tests, initial: $t_{\left(134\right)}=6,\;p<0.001$; replication: $t_{\left(204\right)}=3.5,\;p<0.001$), though here risks preferences did not reverse but rather were nullified in the bandit task. Correspondingly, for all measures of preferences and estimates, participants’ risk preferences better aligned with their estimates in the bandit task, as compared to the SO task (multiple linear regression controlling for task order; all p-values < 0.001 in both the initial study and replication; see Supplementary Table 8 for a full report on the analysis and results).

### Ruling out alternative explanations

To eliminate alternative explanations for the observed differences in risk preferences between the two tasks, we investigated the influence of two key methodological differences between the tasks. First, in the SO task, participants made their choices after viewing *all* chest outcomes, while in the bandit task, choices were intertwined with the learning process, with feedback received after each selection. This difference could potentially introduce a sampling bias in the bandit task, as choices in this task were often made with only partial outcome information, potentially skewing preference patterns. To rule out this sampling bias, we analyzed how choices favoring the high-variance chest evolved over time. If a sampling bias caused the contrasting preference patterns, we would expect those patterns in the bandit task to converge toward the patterns observed in the SO task as participants gained more information. However, this did not occur. Instead, in both initial and replication studies, the reversed preference patterns in the bandit task became more pronounced over time (Figure 3d).

A second methodological difference between the tasks involved the temporal distance between outcome observations and predicted happiness reports, which was greater for the chests reported later in the bandit task. To address this, we tested whether the difference in risk preferences persists when controlling for the chest report order in the bandit task. Multiple linear regression controlling for chest report order (i.e., the sum of both chests’ report order indices) showed that the difference in preference patterns between tasks remained highly significant (multiple linear regression controlling for chest report order; initial: $t_{\left(133\right)}=5.7,\;p<0.001$; replication: $t_{\left(203\right)}=3.5,\;p<0.001$), whereas the effect of report order was insignificant (initial: $t_{\left(133\right)}=0.5,\;p=0.56$; replication: $t_{\left(203\right)}=0.47,\;p=0.64$), indicating the preference pattern differences were not due to a temporal distance.

### Distrbutional learning correlated with a divergence of preferences from estimations

If the observed effect of task on risk preferences was driven by distributional learning, participants whose distributional learning was more strongly affected by the task should show larger changes in risk preferences. To test this prediction, we conducted an exploratory analysis examining the correlation between the task effects on estimation accuracy and on the deviation of risk preferences from estimation biases. The normalized effects on estimation accuracy were averaged, for each participant, over the two kinds of estimations. Task effects on risk preferences were only evident between participants, and thus we averaged each participant’s contributions to the deviation of preferences from estimations (see Supplementary Table 8 and Supplementary Figure 1 for details). We found a weak but statistically significant correlation between the effects of task on estimation accuracy and risk preferences ($r_{p}=0.11,\;t_{\left(369\right)}=2.05,\;p=0.041$). To further test the robustness of this result, we repeated the analysis using a permutation test and after filtering out extreme values in both measures (absolute Z-score > 3). Both approaches yielded similar results (p=0.018 for the permutation test; $r_{p}=0.13,\;t(369)=-2.5,\;p=0.013$ in the parametric test without outliers). Thus, participants who showed more accurate distributional learning in the SO task, as compared to the bandit task, also showed a stronger deviation of preferences from estimations.

### Deviations of preferences from estimations can be explained by a utility function

**Figure 4:**
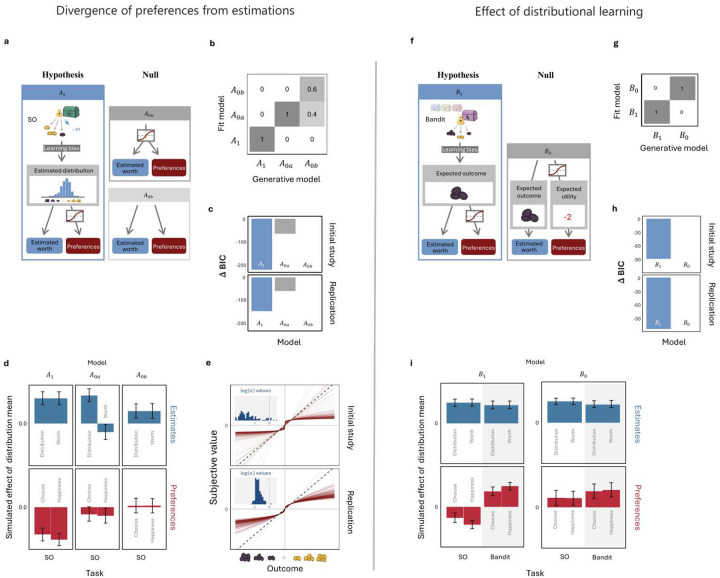
modeling deviation of preferences from estimations We modelled the SO task alone to explain the divergence of preferences from estimations (a-e) and both tasks to explain how this divergence was affected by task (f-i) **a & f. Schematic depiction of candidate models.** The ′A′ models differ on which calculations involve the utility function, whereas the ′B′ models assume different learning mechanisms in the bandit task. Both B models use model $A_{1}$ for the SO task. **b & g. Model recovery.** Confusion matrices presenting the selection rate for each model (based on minimal BIC) for 10 data sets simulated using each of the models. Each model was used to generate experimental data for simulated participants, with the sample size equal to the actual data sample size. Model hyperparameters were fitted to the actual data. We found no confusions between hypothesis and null models. **c & h. Quantitative model comparison.** Bayesian Information Criterion (BIC) is shown for relative to the worst fitting model. The results show that the hypothesized models fitted the data better (i.e., had a lower BIC) than the null models. **d & i. Qualitative model comparison.** Simulated effect on estimation biases and risk preferences of whether outcomes where predominantly positive or negative (‘distribution mean’). Error bars represent 95% confidence intervals. The figure highlights the distinct ability the hypothesized models have for capturing the discrepancy between estimation biases and risk preferences in the SO task $\left(A_{1}\right)$ and its absence in the bandit task $\left(B_{1}\right)$. **e. Fitted utility functions.** This panel presents the utility functions derived from the mean ′a′ parameter fitted for each participant in model $A_{1}$. The histograms display the distribution of the log-transformed ′a′ values used in Eq. 1, while the curves represent the corresponding utility functions, emphasizing individual variation in fitted parameters across participants. The results indicate a strong tendency for negative log-transformed values, which correspond to underweighting of extreme outcomes.

To explain how distributional learning shifts preferences away from estimations, we built a prospect-theory-inspired computational model that predicts both estimations and preferences (Figure 4a). To generate estimates, the model learns outcome distributions from experience. Learning is assumed to be accurate, except that it is allowed to give greater weight to either larger or smaller outcomes and thereby generate an estimation bias. This bias was applied after learning the probabilities for each outcome, using the following equation:

(1) $$p^{\prime }\left(V_{i}\right)=\frac{p\left(V_{i}\right)\cdot e^{\lambda \left|v_{i}\right|}}{\sum_{i}p\left(V_{i}\right)\cdot e^{\lambda \left|v_{i}\right|}}$$

where free parameter λ controls the degree of over or underestimation of the probabilities of extreme outcomes.

To generate preferences, the model translates the objective values of the different possible outcomes into subjective values using a simplified version of the non-linear utility function proposed by Kahneman and Tversky in prospect theory^9^:

(2) $$u(V)=\left\{\begin{array}{cc} V^{a} & \text{for}\;V\geq 0\\ -(-V{)}^{a} & \text{for}\;V<0 \end{array}\right.$$

where V is the raw value (number of coins) and a is a free parameter (a∈(0,∞)) controlling the under / over valuation of large outcomes. These values are then used to calculate the expected utility of each chest, based on the estimated probability of each outcome. Thus, whereas estimations reflect the expected outcome of learned distributions, preferences are based on their expected utility.

To test whether this model explained participants’ estimations and preferences well, we compared it to two null models – one in which the utility function was used both for generating preferences and for estimating expected objective values i.e., estimated worth; $A_{0a}$), and another in which it was used for neither $\left(A_{0b}\right)$. All models were fit to the SO task data, specifically to all task measures used for calculating preferences and estimation biases: the means of estimated distributions, the estimated worths of single sacks, the choices among chests, and the predicted happiness. Model fitting was carried out using an Iterative Importance Sampling algorithm. In both the initial study and the replication study, the hypothesized model outperformed both null models, as indicated by lower Bayesian Information Criterion (BIC) values (Figure 4c).

To ensure that our results were not influenced by specific modelling assumptions, we conducted additional comparisons using two alternative sets of model: one excluding learning biases, and another where estimates were learned using a Rescorla-Wagner learning algorithm^18^ with a constant learning rate, which makes learning affected by observation order (see Methods for details). In both comparisons, the best fitting model used a utility function for generating preferences but not estimates (Supplementary Table 9). Additionally, the constant learning rate model, which is more consistent with non-distributional learning, provided a substantially poorer fit than the model that assumes distributional learning (BICs: 32334 vs. 3143, respectively, in the initial study; and 5030 vs. 4962 in the replication), further supporting the conclusion that participants accurately learned outcome distributions in the SO task. Thus, the divergence of preferences from estimations was well explained by the application of a utility function to learned outcome distributions.

### Utility function explains the effect of distributional learning

To explain why the divergence of preferences from estimations was diminished in the bandit task, where distributional learning was limited, we noted that the effective application of a utility function requires knowing how outcomes are distributed. By contrast, if distributions are reduced to point estimates, the utility function can no longer change preferences between them because it is a monotonic function.

To test this idea, we extended the SO task model to the bandit task, with the only difference being that in the latter task, the model learns only the expected outcome for each chest. Learning takes place using Rescorla-Wagner’s learning rule, while allowing for a learning bias in favor of larger or smaller outcomes:

(3) $$Q_{t+1}=Q_{t}+\eta \left(V_{t}-Q_{t}\right)\;e^{\lambda \left|V_{t}\right|}$$

Where η is fixed learning rate between 0 and 1. The objective estimates, $Q_{t}$, are then converted via a utility function into subjective evaluations, $Q\prime_{t}$, that govern the model’s preferences:

(4) $$Q\prime_{t}=\left\{\begin{array}{cc} {\left(Q_{t}\right)}^{a} & \text{for}\;Q\geq 0\\ -{\left(-Q_{t}\right)}^{a} & \text{for}\;Q<0 \end{array}\right.$$

This conversion results in only minor changes to the relative subjective values of different chests, as the distributional information required for an effective application of the utility function has not been encoded.

We compared this model to a null model $\left(B_{0}\right)$ that effectively applies the utility function in both SO and bandit tasks. This is achieved in the bandit task without distributional learning, by employing two separate learning processes, one for objective estimates (Eq. 3), and one for subjective values:

(5) $$Q\prime_{t+1}=Q\prime_{t}+\eta \left(u\left(V_{t}\right)-Q\prime_{t}\right)\;e^{\lambda \left|V_{t}\right|}$$

A comparison of BIC values showed a clear advantage for our hypothesized model over the null model (Figure 4h). An additional model comparison replacing the constant learning rate in the bandit task with a diminishing learning rate, which weights all observations equally, regardless of their order, returned the same result (see Supplementary Table 10). Notably, the latter models, which are more consistent with accurate distributional learning in the bandit task, fit the data less well than the constant learning rate models, further supporting the assumption of reduced distributional learning in the bandit task. The modeling thus showed how the absence of distributional learning hindered the effective application of a utility function and thus reduced the divergence between preferences and estimations.

### Exploratory analysis of skewness preferences

Preferences between chests associated with positively and negatively skewed outcome distributions were excluded from the preregistered analyses, as they reflected additional factors, such as probability weighting, that go beyond the effects of a utility function. Nevertheless, these preferences also cohered with our hypothesis concerning the role of distributional learning in shaping preferences (Supplementary Figure 2; Supplementary Table 11). Specifically, in the SO task, participants favored positively skewed over negatively skewed chests, as commonly observed in description-based decision tasks. Conversely, in the bandit task, these preferences were eliminated (in predicted happiness measure) or even reversed (in participants’ choices), as commonly observed in experience-based decision tasks. These results further support the possibility that the description-experience gap stems from whether the experimental setup enables participants to acquire full knowledge of outcome distributions rather than from an inherent characteristic of experience-based learning. As discussed in Supplementary Note 1, the effects of compromised distributional learning may include not only a failure to effectively apply the utility function, but also an underweighting of rare events, which is thought to underlie key aspect of the description-experience gap.

## Discussion

We studied the effects of distributional learning on human preferences using a novel dual-task design, with one task designed to facilitate distributional learning and the other designed to hinder it. We validated this novel experimental design using multiple assessments of distributional knowledge, showing that only in the first task, participants’ estimates accurately captured outcome distributions, in both general shape and summary statistics such as mean, variance, and skew. Most importantly, we found that only when distributional learning was facilitated, people showed a qualitative discrepancy between their estimation biases and preferences. Modeling this discrepancy showed that it can emerge due to the application of a utility function in the generation of preferences, which is made ineffective in the absence of distributional learning.

These findings offer important insights into how human preferences are generated. First, the direct comparison between participants’ preferences and their estimations in the SO task demonstrates that preferences in favor of high and low outcome variance are not due to estimation biases, but rather constitute genuine deviation from participants’ own objective estimates of mean outcome. This means that preferences in favor of higher or lower outcome variance are neither a result of distorted probability perception nor a failure to correctly integrate these probabilities. Rather, the most plausible explanation for these preferences is the use of non-linear mapping of outcome to subjective value, as proposed by prospect theory^9^.

The absence of a similar qualitative deviation of preferences from estimations in the bandit task, where distributional learning was compromised, suggests that this deviation depends on distributional learning. Notably, participants observed precisely the same outcomes in the two tasks, except that in the bandit task, outcomes from different distributions were interleaved, making it difficult for participants to learn the entire shape of the distributions. Our model demonstrated how the effect of impaired distributional learning on preferences may emerge: learning how outcomes are distributed enables the application of a utility function to different potential outcomes, so as to compute the expected utility. In the absence of information about different potential outcomes, the utility function can only be applied to the expected outcome, and therefore would not affect preferences.

Notably, distributional learning is only necessary if the utility function is applied to learned information at the time of decision. An alternative model could apply the utility function to each individual outcome at the time of experience, such that an expected utility is learned directly, without distributional learning. This latter model, however, did not fit with participants estimations and preferences in the present study. Given the assumption that human learning should be geared towards decision-making, it may seem unreasonable that humans do not directly learn expected utilities, but rather learn expected outcomes and only transform them into utilities. However, at least in some settings, this assumption may be incorrect. Indeed, a wide range of evidence suggests that human learning is primarily geared towards building accurate probabilistic models of the environment^19,20^. This motivation may be particularly enhanced in our task because it requires participants to report their estimates. When such learning is successful, it enables more flexible evaluations and better decision-making. However, when impaired, the agent is left with a point estimate of the expected outcome, which it cannot translate into expected utility.

Our findings resonate with prior work on the description-experience gap^13,15^, replicating differences in risk and skewness preferences, and suggesting an additional factor contributing to this gap: the effective application of a utility function. Importantly, our results demonstrate that this gap should not be attributed to the method of information acquisition, as it is not linked to learning from description versus experience per se, but rather to the quality of distributional learning. Learning from description is merely one instance where participants can successfully learn the outcome distribution. Thus, we propose an “experience–experience gap”, whereby risk preferences differ across different instances of learning from experience: under simple learning conditions, people show the same risk preferences that are commonly observed in description-based tasks, but when learning from experience is made more challenging, risk preferences shift towards patterns more commonly observed in experience-based studies^13,15^. Therefore, what is learned, rather than how it is learned, may be the key factor shaping risk preferences across different settings.

More generally, our finding that participants’ preferences can deviate not only from the expected outcome of the true distribution but also from their own objective evaluations reinforces the distinction between objective and subjective values, recognized since the introduction of utility functions. This emphasizes the limitations of inferring preferences from estimations, or vice versa, and thus calls for a more careful interpretations of prior work that only assessed one or the other. With regards to future work, our findings underscore the importance of differentiating between objective and subjective evaluations in learning and decision-making studies, as these can differ significantly by task. The present work demonstrates how this differentiation can be achieved in practice.

An interesting implication of the observed divergence between preference and estimates is that people can derive different evaluations from the same estimates. In the present study, we demonstrated this by having participants generate both objective and subjective evaluations. However, once agents maintain estimates and preferences separately, they could theoretically generate several distinct subjective evaluations from the same estimated outcomes. Such distinct evaluations could arise from differences in how different learning or decision-making systems calculate subjective value^21,22^. This may have important consequences for understanding how internal conflicts drive conflicting behaviors and emotional responses^23^.

Our experiment compared only two tasks. Future research which incorporates a broader range of tasks, manipulating learning difficulty by different methods, could more definitively test the effects of distributional learning. Additionally, testing a broader set of distributions could enable a more detailed characterization of how distributional learning is compromised and how this affects preferences, including between distributions that differ by more than one statistic.

In sum, we identified a systematic divergence of risk preferences from estimation biases, and showed how it may emerge because the effective application of a utility function requires information about different potential outcomes. Our findings thus indicate a key role for distributional learning in shaping human preferences and reveal how and when preferences deviate from normative models of decision making.

## Methods

### Participants

We recruited 423 participants (179 in the initial study and another 244 for the replication study) using the Prolific online participant recruitment platform. Participants completed a 35-minute online task for which they were compensated with £5.3 to £8.3 depending on their performance. Participants were aged between 20 and 50 years (M = 36, SD = 11), with a balanced gender distribution (51% male, 46% female, 3% preferred not to disclose their gender). All participants were native English speakers from the United States. 52 participants (initial – 19, replication – 33) were excluded based on quality criteria (preregistered for the replication study at https://osf.io/vq2hc). Based on these criteria, we also omitted several participants from specific analyses. The exclusions and exclusion criteria are detailed below.

### Procedure

The study comprised a sequential observation task and a bandit task, with task order randomized across participants. In each of these tasks, participants observed an equal number of outcomes drawn from each of four chests. To increase engagement, participants first chose the treasure chests they would encounter in each task, but these choices only affected the visual appearance of the chests.

### Sequential Observation task

Participants observed the numbers of gold or dark coins contained in sacks drawn from a chest one at a time. Gold coins represented positive amounts, up to +7, and dark coins represented negative amounts, up to −7. After observing 30 sacks, participants reported their estimates and emotional evaluations for that chest and then proceeded to the next chest. After completing this procedure for four chests, participants made 18 choices, each time choosing between two sacks drawn from two different chests. In this way, participants chose between each pair of chests three times. The contents of the sacks were not revealed following participants’ choices, but participants were informed that their choices would affect their bonus.

### Bandit task

Four chests were presented side by side on the screen. In each round, two sacks were drawn from two different chests, and the participant chose one. The outcomes of both the chosen and unchosen sacks were briefly displayed, followed by two new sacks drawn from two new chests. After 60 rounds (30 sacks per chest), participants reported their estimates and emotional evaluations for all four chests sequentially, starting with the leftmost chest and moving to the right.

### Outcome distributions

Each task’s four chests were randomly assigned one of four outcome distributions: symmetric with high variance, symmetric with low variance, positively skewed, or negatively skewed. The distributions are presented in figure 1b. For each given participant, either the symmetric distributions were both centered around a negative mean outcome of −2 coins and the skewed distributions were centered around a positive mean outcome of +2 coins, or vice versa.

### Estimated distribution

Participants estimated the number of times each possible outcome would occur if 100 more sacks were drawn from each treasure chest using an array of sliders.

### Estimated worth

Participants estimated the worth of a single sack drawn each chest, the best of 5 sacks drawn from the chest, and the worst of 5 sacks. They reported their estimates on a single slider ranging from −7 to +7 coins, with separate handles for each type of estimate.

### Predicted emotional response

Participants were instructed to imagine a scenario where their payment for the study corresponded to the worth of a random sack drawn from the chest, with each coin representing a gain or loss of $100. They reported using a slider how happy they would be if this were the case. For exploratory purposes, we also asked participants to report how anxious and excited they would feel before discovering the contents of the sack.

### Exclusion criteria

For each participant, we calculated the extent to which they favored chests with a high mean outcome over those with a low mean outcome, in each of the tasks. Specifically, we considered (1) the mean difference between the expected values of estimated distributions for high- and low-mean-outcome chests, (2) the difference in the estimated worth of a single sack drawn from these chests, (3) participants’ choices between chests, and (4) the difference in predicted happiness when payment was supposedly based on the outcomes of sacks drawn from chests with high vs. low mean outcomes.

We then normalized this calculation into sensitivity scores for the objective reports (estimated distribution and worth), such that 0 represented chance performance and 1 represented the score for an ideal observer. Participants with a sensitivity score below 0.25 on either measure were excluded, resulting in the removal of 19 participants in the initial study (estimated distribution: 2; estimated worth: 10; both: 7) and 29 participants in the replication study (estimated distribution: 3; estimated worth: 14; both: 12). Participants who failed a single attention check embedded in the questionnaires were also excluded (initial: 0; replication: 4).

Additionally, participants with a sensitivity score below 0 in choices or predicted happiness—indicating a preference for lower-mean-outcome chests—were excluded from analyses involving these measures. This exclusion affected 11 participants in the initial study (choices: 8; happiness: 5; both: 1) and 19 in the replication study (choices: 15; happiness: 2; both: 2).

### Questionnaires

For exploratory purposes, we had participants answer several self-report personality questionnaires at the end of the study. These included the trait anxiety part of the State Trait Anxiety Inventory (STAI)^24^, the Temperament Evaluation of Memphis, Pisa, Paris and San. Diego (TEMPS A)^25^, a brief version of the Barratt Impulsivity Scale (BIS)^26^, and the General Risk Propensity Scale (GRiPS)^27^.

### Computational models

Computational models were fit to the data using an iterative importance sampling algorithm^28–30^ implemented in python, which has been demonstrated to achieve high parameter and model recoverability^25^. Each iteration, we drew 10,000 independent parameter samples from group-level distributions, weighted each sample by its likelihood given the data, and then fitted the group-level hyperparameters to the weighted distribution of samples. This was repeated until convergence. The models were developed and tested on data from the initial study and then specified in a preregistration prior to the replication study.

To explain the discrepancy between estimates and preferences in the SO task, we assumed agents learn the distribution of outcomes accurately, with an optional learning bias allowing the models to over or underweight extreme outcomes (Eq. 1). These probabilities were then used to calculate the expected outcome and utility, with utility values calculated using a simplified version of the utility function suggested in prospect theory (Eq. 2). The simplified version omitted a loss aversion parameter in order to avoid overfitting. This was required due to the small number of observations per participant, and the absence of outcome distributions that comprise comparable numbers of positive and negative outcomes.

We then used the learned expected outcomes to predict, for each chest, the expected value of the participant’s estimated distribution and their estimated worth for a single sack drawn from the chest. Similarly, we used the expected utility to predict choices and predicted happiness ratings.

The likelihood for estimates and happiness ratings was calculated using a normal distribution—with the relevant learned value as the mean and a dedicated free parameter for variance. Since the null models (described shortly) incorporated the utility function in predicting estimation measures—potentially distorting the variance of learned values— we scaled both learned values and reported estimates to a standard deviation of one. Predicted happiness values were similarly scaled and mean-centered to remove individual differences in how participants used the rating scale, both in terms of mean happiness and variability relative to changes in expected utility. The likelihood for choices was calculated by applying a logistic function to the difference in expected utility between the two available chests, multiplied by a free inverse temperature parameter.

We compared this hypothesized model $\left(A_{1}\right)$ to two null models. The first $\left(A_{0a}\right)$ used the utility function to both generate preferences and calculate estimated worth. Estimated distribution remained unaffected, as it did not involve evaluation. The second null model $\left(A_{0b}\right)$ relied solely on probability estimates, without applying a utility function to generate estimates or preferences.

We ran two additional versions of this comparison in order to rule out the effects of our modeling assumptions – one with no attention bias (λ=0), and another in which the models did not learn the whole distribution of outcomes but rather maintained two separate point estimates which were updated throughout observation:

(6) $$Q_{t+1}=Q_{t}+\eta \left(V_{t}-Q_{t}\right)$$

(7) $$Q\prime_{t+1}=Q\prime_{t}+\eta \left(u\left(V_{t}\right)-Q\prime_{t}\right)$$

With Q representing the objective evaluation and $Q^{\prime }$ the subjective evaluation.

To determine whether the effect of task on the divergence between estimates and preferences could be attributed to distributional learning, we extended the hypothesized model of the SO task to the bandit task. To correspond with the observation of reduced distributional learning in the bandit task, we modeled learning as an update to a point estimate of the expected outcome (Eq. 3). Utility values were therefore calculated by applying the utility function to point estimates (Eq. 4), which minimizes the impact of the utility function on preferences.

The null model, in contrast, assumed that subjective evaluation occurs when observing outcomes. Thus, both objective (Eq. 3) and subjective (Eq. 5) evaluations are maintained and updated in parallel throughout the task.

As with the first model comparison, we tested an additional model set to ensure our results were not solely a consequence of particular modeling assumptions. For this comparison, we considered models that did not assume a constant learning rate for the bandit task. Instead, we modeled the learning rate as decreasing over time, similar to equally averaging all previously observed outcomes. This model also incorporated the same attention bias defined earlier:

(8) $$Q_{t+1}=\frac{Q_{t}*W_{t}+V_{t}*e^{\lambda \left|V_{i}\right|}}{W_{t}+e^{\lambda \left|V_{i}\right|}}$$

where $W_{t}$ represents the sum of weights accumulated until round t:

(9) $$W_{t+1}=W_{t}+e^{\lambda \left|V_{i}\right|}$$

The update function for the subjective evaluation $\left(Q^{\prime }\right)$ in $B_{0}$ used the same formula, but with values converted via the utility function:

(10) $$Q\prime_{t+1}=\frac{Q\prime_{t}*W_{t}+u\left(V_{t}\right)*e^{\lambda \left|V_{i}\right|}}{W_{t}+e^{\lambda \left|V_{i}\right|}}$$

Another tested model $\left(B_{2}\right)$ assumed that the differences between tasks are due to an increased learning bias in the bandit task which canceled out the effects of the utility function. This model fitted the data slightly less well than the hypothesized model (BIC = 2677 in initial study and 4824 in replication). We did not include this model in the main results section because it cannot qualitatively explain the reduced divergence between preferences and estimates in the bandit task.
